# The Ability of a Non-Egg Adapted (Cell-Like) A(H1N1)pdm09 Virus to Egg-Adapt at HA Loci Other than 222 and 223 and Its Effect on the Yield of Viral Protein

**DOI:** 10.1371/journal.pone.0166761

**Published:** 2016-11-18

**Authors:** Carolyn Nicolson, Ruth Harvey, Othmar G. Engelhardt, James S. Robertson

**Affiliations:** National Institute for Biological Standards and Control, MHRA, Blanche Lane, South Mimms, Potters Bar, Hertfordshire, EN6 3QG, United Kingdom; University of South Dakota, UNITED STATES

## Abstract

Previous studies on influenza A(H1N1)pdm09 candidate vaccine viruses (CVVs) that had adapted to growth in embryonated chicken eggs by the acquisition of amino acid substitutions at HA positions 222 or 223 showed that improved protein yield could be conferred by additional amino acid substitutions in the haemagglutinin (HA) that arose naturally during passaging of the virus in eggs. In this study we investigated, by means of reverse genetics, the ability of a non-egg adapted (cell-like) A(H1N1)pdm09 virus to egg-adapt at HA loci other than 222/223, introducing amino acid substitutions previously identified as egg adaptations in pre-H1N1pdm09 H1N1 viruses and assessing their effect on protein yield and antigenicity. We also investigated the effect on the protein yield of these substitutions in viruses that had A(H1N1)pdm09 internal genes rather than the traditional PR8 internal genes of a CVV. The data show that a cell-like A/Christchurch/16/2010 can be egg-adapted via amino acid substitutions in at least three alternative HA loci (187, 190 and 216), in viruses with either PR8 or A/California/7/2009 internal genes, but that the effects on protein yield vary depending on the amino acid substitution and the internal genes of the virus. Since CVVs need to produce high protein yields to be suitable for vaccine manufacture, the findings of this study will assist in the future characterisation of both wild type viruses and lab-derived CVVs for vaccine use.

## Introduction

Influenza is an annual global threat to public health, mitigated by seasonal vaccination. Due to antigenic drift of influenza viruses, there is a need to continually assess features of the viruses causing disease and, when necessary, update the virus strain(s) being used to manufacture vaccine. Each new candidate vaccine virus (CVV) needs to be adapted to growth in embryonated hens’ eggs, whilst maintaining the appropriate antigenicity. Such egg-adapted viruses generally have amino acid substitutions around the receptor binding site of the haemagglutinin (HA) that can impact on viral attributes such as antigenicity and growth [1, 2].

A(H1N1)pdm09 viruses continue to circulate and are included in seasonal influenza vaccine. It is expected that H1N1 virus will undergo antigenic drift and new egg isolates will be required for inclusion in future seasonal vaccines. This study was conducted to investigate various substitutions that could lead to egg-adaptation of the H1N1 virus and the impact of these on antigenicity, growth of virus and yield of antigen. A comparison of HA structures showed that known egg adaptation loci of pre-H1N1pdm09 viruses map to the same positions around the receptor binding site in H1N1pdm09 viruses. In absence of other information on egg-adaptive mutations of H1N1pdm viruses, it was logical to assess the pre-2009 egg-adaptive sites. Therefore known substitutions that occurred during egg-adaptation of A(H1N1)pdm09 viruses [1], and those that occurred during egg-adaptation of pre-H1N1pdm09 H1N1 viruses [3,4] at these equivalent loci, were introduced into a non-egg adapted (cell-like) A/Christchurch/16/2010 HA gene using reverse genetics.

Paired viruses were generated with both PR8 or A/California/7/2009 internal genes and the effect of the HA substitutions on viruses resembling either laboratory derived CVVs or naturally occurring viruses assessed.

## Material and Methods

### Viruses

All viruses were engineered from A/Christchurch/16/2010 (Chr/16) A(H1N1)pdm09, which is antigenically equivalent to A/California/7/2009 (Cal/7), the A(H1N1)pdm09 reference strain, despite differing from it at passage E2 by 9 amino acid residues in HA (in addition to the 222,223 motif which is mixed in Cal/7) and 6 residues in neuraminidase (NA). [Chr/16 GISAID number EPI_ISL_79239; HA EPI278607, NA EPI278608].

### Virus growth

Viruses were grown in 10 or 11 day old embryonated hens’ eggs at a dilution of 10^−5^ in PBS’A’ at 35°C for 72 hours. Allantoic fluid was harvested from chilled eggs and virus growth was assessed using the haemagglutination (HA) assay following standard protocols with 0.7% turkey RBC. Embryonated hens’ eggs used in this study were exempt from ethical approval as they were not within the final third of their incubation period (Animals (Scientific Procedures) Act 1986 Amendment Regulations 2013).

Viruses were grown in MDCK cells, in MEM/1% L-glutamine/1.5% HEPES/2% bovine albumin and 2.5μg/ml TPCK trypsin (Worthington), according to standard protocols. Cells were incubated at 35°C/5%CO_2_ for 72 hours. Cell medium was clarified at 1500rpm/5mins/4°C in a JS5.3 (Avanti) rotor and virus growth assessed by HA assay.

### Generation of recombinant viruses

Mutations were introduced into the HA gene of A/Christchurch/16/2010 as shown in Table 1 using recombinant PCR and cloning techniques as described [5]. Residues 119 and 129 have been implicated previously in egg adaption of H1N1pdm09 viruses, whilst N, V and A substitutions at 187 reflect the egg adaptation residues found at the equivalently positioned 190 residue in pre-H1N1pdm09 viruses. A substitution of L at 186 reflects the egg adaptation residue found at the equivalently positioned 189 residue in pre-H1N1pdm09 viruses, whilst a D at 186 has been found to increase growth of H1N1pdm09 viruses in cells and eggs albeit in a virus already egg adapted at another loci.

**Table 1:** Genetic stability of potential egg-adaptive amino acid substitutions introduced into the HA gene.

HA amino acid substitution^5^	HA sequence at E6	Additional HA substitution at E6
-^1^	-	I216R
K119N	119N	S190R
N129D^2^	129D	Q223R
A186L	186A rev^3^	D187S
A186D^2^	186D	Q223R
D187N	187D rev^3^	190 S/R; 222D/N^4^
D187V	187V	-
D187A	unsuccessful rescue	-

^1^ DQ HA gene of A/Christchurch/16/2010

^2^ viruses required a single passage in MDCK cells before successful passaging in eggs (i.e. M1E6)

^3^ ‘*rev*’ denotes a reversion to the original amino acid in the wt A/Christchurch/16/2010 HA gene

^4^ mixed sequence at these positions

^5^ Residues 119 and 129 have been implicated previously in egg adaption of H1N1pdm09 viruses. N, V and A substitutions at 187 reflect the egg adaptation residues found at the equivalent 190 residue in pre-swH1 viruses. D at 186 has been found to increase growth of H1N1pdm09 viruses in cells and eggs but in viruses already egg adapted at other loci. An L substitution at 186 reflects the egg adaptation residue found at the equivalent 189 residue in pre-H1N1pdm09 viruses.

Viruses were derived using these HA genes and the NA gene from A/Christchurch/16/2010, using reverse genetics as previously described [5]. A total of 8 viruses were derived using these mutated HA genes from Chr/16, whilst another 2 viruses were also derived by reverse genetics with non-mutated HA genes (Table 2). Viruses were passaged in eggs six times as above and their HA and NA genes sequenced using ABI Dye termination method and a Gene Scanner 3130.

**Table 2:** Panel of viruses created and used for yield studies.

Viruses with PR8 internal genes	Viruses with H1N1pdm09 internal genes	HA position 222/223^4^	Additional amino acid substitutions introduced into the HA^4^
NIB-74xp^1^	Chr/16 (wt)	NQ	-
RG255^2^	RG260^3^	NQ	-
RG259^2^	RG263^3^	DQ	I216R
RG258^2^	RG262^3^	DQ	S190R
RG257^2^	RG261^3^	DQ	D187S
RG236^2^	RG264^3^	DQ	D187V

^1^ classical reassortant virus derived from a co-infection with A/Christchurch/16/2010 (Chr/16) and NYMC X-157 (the latter providing PR8 internal genes to NIB74xp)

^2^ the six internal genes are derived from cloned PR8 used in reverse genetics, the sequences of which differ from NIBSC PR8 virus stock

^3^ internal genes derived from A/California/7/2009

^4^ sequences were determined at passage level E6, except for NIB-74xp (passage E14) and A/Christchurch/16/2010 (passage E4)

### Haemagglutination inhibition assay

Viruses were assayed in a haemagglutination inhibition (HI) assay according to standard protocols [6] using 0.7% turkey RBC. Each virus was assayed at 8 agglutinating doses against ferret antiserum raised against NIB-74xp E14 and A/California/7/2009 wt. Only one virus was tested for each HA mutation introduced as a change in the internal genes would not affect antigenicity of the HA.

### Virus concentrates

Virus concentrates were prepared from batches of 30 eggs as described in [2]. Final virus pellets were resuspended in 200μl PBS‘A’ and stored at 4°C. Three separate concentrates were prepared for each virus, with NIB-74xp grown alongside each as a control.

### Total protein assay

Total protein content of virus concentrates was assayed using a BCA assay kit (Pierce) according to manufacturer’s instructions.

### SDS PAGE and deglycosylation

SDS-PAGE analysis including deglycosylation was carried out as previously described [7]. Quantitation was carried out using an ImageScanner with associated ImageQuant software (GE Healthcare).

### SRD

Single radial diffusion (SRD) assays were performed as previously described [8]. Virus concentrates were measured against NIB-74 reference antigen (NIBSC code 10/258) using sheep antiserum raised against A/California/7/2009 (NIBSC code 12/108). Zones were measured using a Synoptics Image Analyser and comparisons were performed using Combistats analysis software, v4.0 (EDQM).

### Infectivity (EID50/ml)

Infectivity assays were performed as previously described [2]. The infectivity titre expressed as EID50/ml was calculated using the Reed and Muench method [9,2,7].

### Statistical analysis

Statistical analysis was performed using Minitab 16 software. Analysis of variance (ANOVA) was performed using the general linear model with the Tukey method for pairwise comparisons and 95% confidence limits [10].

## Results

### HA constructs

This study uses (H1N1)pdm09 numbering. Substitutions at HA residues 222 or 223 have been shown previously to be associated with egg adaptation and in non-egg adapted virus these positions are expected to be 222D, 223Q. The HA gene of wt egg adapted A/Christchurch/16/2010 (with egg adaptation 222N) was engineered to contain the ‘cell-like’ residue 222D [1], and is subsequently referred to here as a DQ HA. The amino acid residues chosen for substitution in the DQ HA were those previously associated with egg adaptation without affecting antigenicity: residues 186 and 187 [3,4] and residues 119 and 129, identified as conferring high protein yields in A/California/7/2009 [1,2].

#### Genetic stability of amino acid substitutions

A panel of viruses with PR8 internal genes was assessed for the genetic stability of potential egg adaptive substitutions introduced into a DQ HA gene (Table 1). Viruses were egg passaged six times (E6) and their HA and NA genes sequenced. No changes were found in the NA gene (data not shown). Only one virus, D187V, remained genetically stable at E6. All other viruses acquired one or more additional HA mutations whilst two of them, A186L and D187N, also reverted the introduced mutation. Viruses N129D and A186D required a single passage in MDCK cells before successful growth in eggs and both acquired Q223R during subsequent egg passage. The DQ HA control virus maintained cell-like 222D/223Q but acquired I216R by E6.

We concluded from these data that HA amino acid substitutions I216R, D187S and D187V were single-site egg adaptations compatible with the cell–like 222D/223Q motif. We also concluded that S190R may also be compatible with the cell like 222D/223Q motif and therefore should be included in the viruses created for yield studies.

#### Viruses created for yield studies

HA genes were constructed containing 222D/223Q plus amino acid substitutions identified from the genetic stability data. They were rescued along with A/Christchurch/16/2010 NA gene and the six internal genes of either PR8 or the A(H1N1)pdm09 reference virus A/California/7/2009 (Table 2). In addition, reverse genetics analogues of NIB-74xp (NIBRG-255) and A/Christchurch/16/2010 wt (A/Christchurch/16/2010 HA and NA on a A/California/7/2009 backbone) (NIBRG-260) were constructed. These new viruses were egg passaged six times (E6) and their HA and NA genes sequenced to confirm genetic stability. No further minor populations were found during this sequencing. The classical reassortant NIB-74xp, containing the HA and NA genes of A/Christchurch/16/2010 and the six internal genes from PR8, as well as wt virus A/Christchurch/16/2010 were included in the yield studies.

### Yield studies

**Fig 1:**
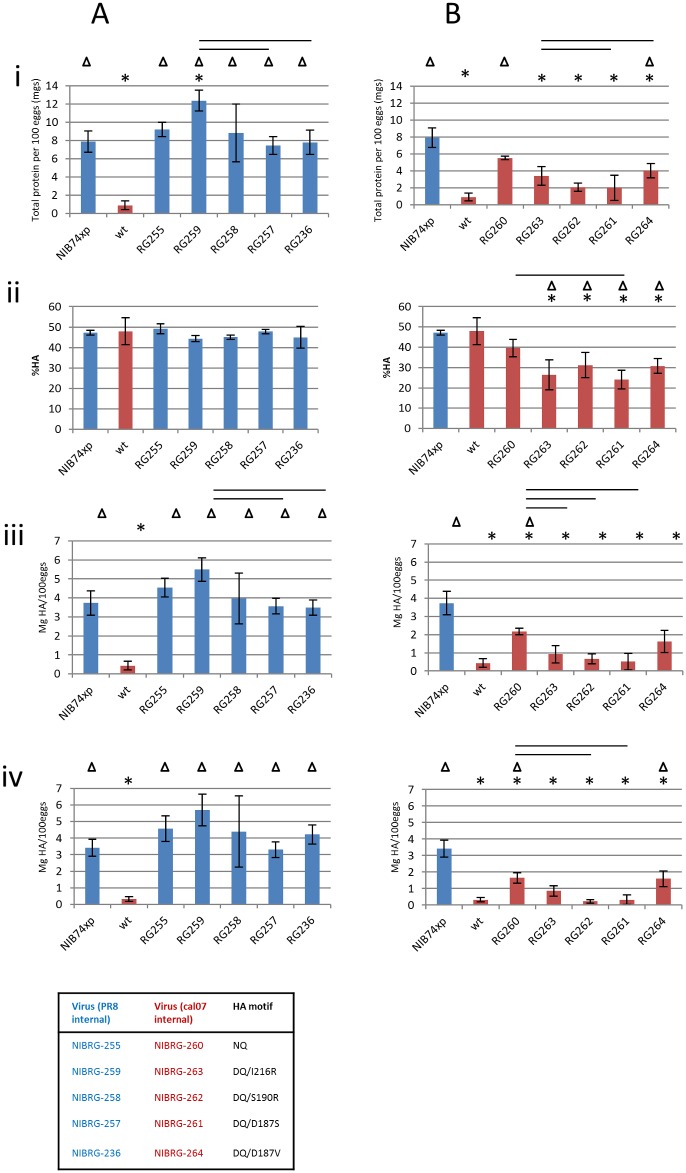
Yield analysis of viruses. Panel A shows data for viruses with PR8 internal genes (in blue) compared to Chr/16 wt (in red). Panel B shows data for viruses with A(H1N1)pdm09 internal genes (in red) compared to NIB-74xp (in blue). In each panel the asterisks (*) denote statistically significant differences to NIB-74xp (p<0.05), and a triangle (Δ) denotes a statistically significant difference to Chr/16 wt (p<0.05). The black bars denote statistically significant differences (p<0.05) between the other viruses. (i) Total protein yield for each virus measured by BCA assay. (ii) HA content of each virus, expressed as percentage HA relative to the total of the four major viral protein bands (HA1, HA2, NP, M1), as measured by SDS-PAGE analysis. (iii) Average yield of total HA protein calculated from percentage HA and total protein concentration data. (iv) Average yield of HA as measured by SRD assay. All data are the average of at least three independent experiments and error bars denote standard deviation.

**Fig 2:**
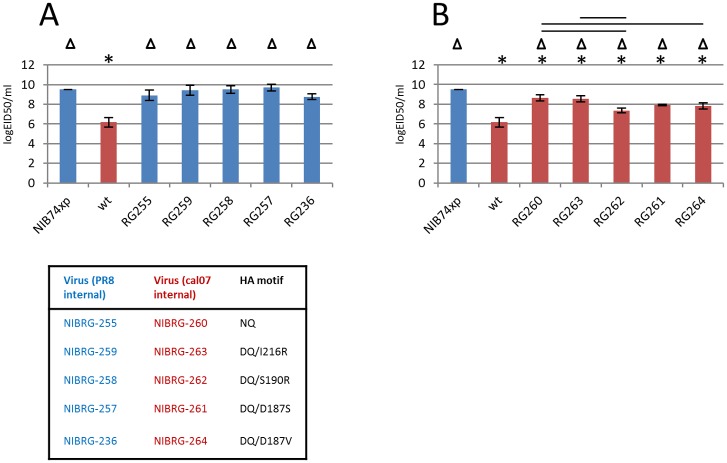
Infectivity of viruses. EID50 assays were performed and the results calculated as described in materials and methods. Panel A shows data for viruses with PR8 internal genes (in blue) compared to Chr/16 wt (in red). Panel B shows the data for viruses with A(H1N1)pdm09 internal genes (in red) compared to NIB74xp (in blue). In each panel the asterisks (*) denote a statistically significant difference to NIB74xp (p<0.05), and a triangle (Δ) denotes a statistically significant difference to Chr/16 wt (p<0.05). The black bars denote statistically significance differences (p<0.05) between the other viruses. Values shown are the average of three independent experiments and error bars show standard deviations.

Total viral protein yield, %HA content, mgs HA protein and mgs functional HA protein are shown in Fig 1. Panel A shows data for viruses with PR8 internal genes (in blue) compared to Chr/16 wt (in red); panel B shows data for viruses with A(H1N1)pdm09 internal genes (in red) compared to NIB-74xp (in blue). Fig 2 shows infectivity data.

#### Yield of viruses with PR8 internal genes

All viruses with a PR8 backbone had higher yield (as measured by total protein and HA yield) than viruses with an A(H1N1)pdm09 backbone, irrespective of the HA sequence (blue bars, Fig 1, Panels A and B, i,iii,iv). The same pattern of yield across panels of viruses is observed in all yield assays (Fig 1, panels i,iii,iv). Similarly, HA content of viruses with PR8 backbone is higher than that of viruses with A(H1N1)pdm09 backbone (Fig 1, Panels A and B, ii), except for NIB-74xp and Chr/16, which have equivalent HA content.

There is no significant difference (p>0.05) between viruses with 222N/223Q but different sources of PR8 internal genes (NIB-74xp with NIBSC PR8 versus NIBRG-255 with Oxford PR8 internal genes) by any measure of yield (Fig 1).

Fig 1, Panel A (i) shows that the total protein yield of all viruses with a PR8 backbone (blue bars) was significantly higher than Chr/16 (NQ; red bar) with yields of 7-12mg total protein/100 eggs compared to 1mg total protein/100 eggs (p<0.05). NIBRG-259 (DQ/I216R; 12mg) is significantly different to NIB-74xp (8mg), NIBRG-257 (DQ/D187S; 7.5mg) and NIBRG-236 (DQ/D187V; 8mg) (p<0.05), but not to NIBRG-255 (NQ; 9mg) or NIBRG-258 (DQ/S190R; 9mg).

There was no significant difference in HA content across the panel of viruses. Thus the HA yield (Panel A, iii) shows a similar trend to that of total protein yield although not all differences were statistically significant. NIBRG-259 had the highest yield (5.5mg HA/ml) and was statistically significantly different to NIBRG-257 and NIBRG-236 (~3.5mg HA/ml each). The yield of functional HA of PR8 backbone viruses, as measured by SRD (Panel A, iv), ranges from ~3.5mg HA/ml (NIB-74xp) to ~5.5mg HA/ml (NIBRG-259) with no significant differences between them. Their SRD HA yield however was significantly different to that of wt Chr/16 (0.5mg HA/ml). The data show that when PR8 internal genes are present, the yield of 222D/223Q viruses is equivalent to, or even higher than the 222N/223Q viruses (depending on alternative substitutions).

#### Yield of viruses with A(H1N1)pdm09 backbones

Viruses with A(H1N1)pdm09 derived internal genes gave much lower protein yields in all assays compared to viruses with PR8 internal genes, irrespective of the 222/223 motif or other HA substitutions (red bars, Fig 1, Panels A and B, i,iii,iv). In contrast to viruses with a PR8 backbone, viruses with an A(H1N1)pdm09 backbone show variable and significantly less HA content compared to NIB-74xp and Chr/16 wt which have an equivalent HA content (Panel B, ii), a measurement independent of virus yield.

Viruses with an NQ HA and either Chr/16 backbone (wt A/Christchurch/16/2010) or Cal/7 backbone (NIBRG-260), are significantly different to each other (p<0.05) in total protein and HA yield (Panel B, i,iii,iv), implying that the presence of different A(H1N1)pdm09 backbones affects yield.

Fig 1 (Panel B, i) shows that NIBRG-260 (5mg/100 eggs; NQ motif) was significantly different to Chr/16 (1mg), NIBRG-262 (2mg; DQ/S190R) and NIBRG-261 (2mg; DQ/D187S), but not to NIB-74xp (8mg), NIBRG-263 (3mg; DQ/I216R) and NIBRG-264 (3mg; DQ/D187V). All DQ viruses were not significantly different to each other.

For HA protein yield, DQ viruses range from ~0.5mg/100 eggs (NIBRG-261) to 1.5mg/100 eggs (NIBRG-264); however, these four viruses are not statistically significantly different from Chr/16 (p>0.05). NIBRG-260 is significantly different from both wt Chr/16 and NIB-74xp (3.5mg). These data reflect those for total protein yield.

The pattern of yield of functional HA protein for these viruses (Fig 1, Panel B, iv) is also similar to those mentioned above. Wildtype Chr/16, NIBRG-262 and NIBRG-261 all have <0.5mg/ml SRD HA whilst NIBRG-263 has ~1mg/ml. These four viruses are not significantly different from each other but are significantly different from NIB-74xp (3.5mg). Viruses NIBRG-260 and NIBRG-264 (both ~1.75mg) are significantly different to both NIB-74xp and Chr/16 wt.

Fig 1 (Panel B, ii) shows that Chr/16 (NQ) and NIB-74xp (NQ) do not differ significantly in their HA content (both ~47%), but inclusion of a Cal/7 backbone in NIBRG-260 (NQ) reduces the HA content to 40%; a DQ motif reduces it further to 30% or below. All DQ viruses are significantly different to NIB-74xp and Chr/16 wt. NIBRG-261 (DQ/D1867S) has the lowest HA content (25%) but is significantly different only to viruses with the NQ motif (wt Chr/16, NIB-74xp and NIBRG-260). Therefore whilst a Cal/7 backbone improves the protein yield of Chr/16 it also reduces the HA content. This reduction is compounded by replacement of the NQ motif with DQ plus additional amino acid substitutions.

### Infectivity

Infectivity titres are shown in Fig 2. For viruses with a PR8 backbone (blue bars, Panel A) the infectivity titres are largely comparable (10^9^ to 10^9.5^) between different HAs, whilst the titre for wt Chr/16 is 3 logs lower (10^6^ EID50/ml, red bar). Titres of all PR8 backbone viruses are not significantly different from each other but are significantly different to wt Chr/16 (p<0.05).

Viruses with an A(H1N1)pdm09 backbone (red bars, Panel B) show a wider variation in infectivity, from 10^6^ for wt Chr/16 (with its own internal genes) to 10^8.5^ for NIBRG-260 and NIBRG-263 (with Cal/7 internal genes). Titres for Chr/16 and NIB-74xp are significantly different, reflecting their 3 log difference in infectivity, with each of them significantly different to all other viruses in the data set. Of the remaining viruses, NIBRG-260 (NQ; 10^8.5^) is significantly different to NIBRG-262 (DQ/S190R; 10^7^) and NIBRG-264 (DQ/D187V; 10^7.5^), but not to NIBRG-263 (DQ/I216R; 10^8.5^) and NIBRG-261 (DQ/D187S; 10^8^), whilst NIBRG-262 is also significantly different to NIBRG-263. The data show that replacing the wt Chr/16 backbone with a Cal/7 backbone increases the infectivity of a ‘wt-like’ NQ virus, and that further replacement of the NQ motif with a DQ motif can result in equivocal infectivity depending on the additional egg adaptive amino acid substitution.

### Antigenicity

A Haemagglutination inhibition assay was used to assess potential antigenic changes conferred by the amino acid substitutions; all viruses had HI titres within twofold of the titres obtained with wt Chr/16 and so it was concluded that there were no antigenic differences (Table 3).

**Table 3:** Antigenic analysis of viruses by haemagglutination inhibition assay.

	Ferret Sera^1^	
Viruses	F12/09	F149/11
NIBRG-255	1280	1280
NIBRG-257	640	1280
NIBRG-258	320	1280
NIBRG-259	640	2560
NIB-74	640	2560
NIB-74xp	640	**2560**
A/Christchurch/16/2010	640	2560

^1^ Ferret sera were raised against viruses as follows: F12/09 –A/California/7/2009; F149/11 –NIB-74xp

## Discussion

In the development of seasonal candidate vaccine viruses (CVVs) for egg vaccine production, viruses undergo natural selection in the egg although some of the amino acid substitutions selected for in eggs can affect the viruses’ antigenicity. However, the impact of egg-adaptive substitutions on yield, another important phenotype of CVVs, is not well understood. This study was designed to assess the impact of a variety of egg-adapting mutations, introduced rationally by reverse genetics, on the protein and HA yield, and the infectivity, of seasonal H1N1pdm09 CVV.

**Fig 3:**
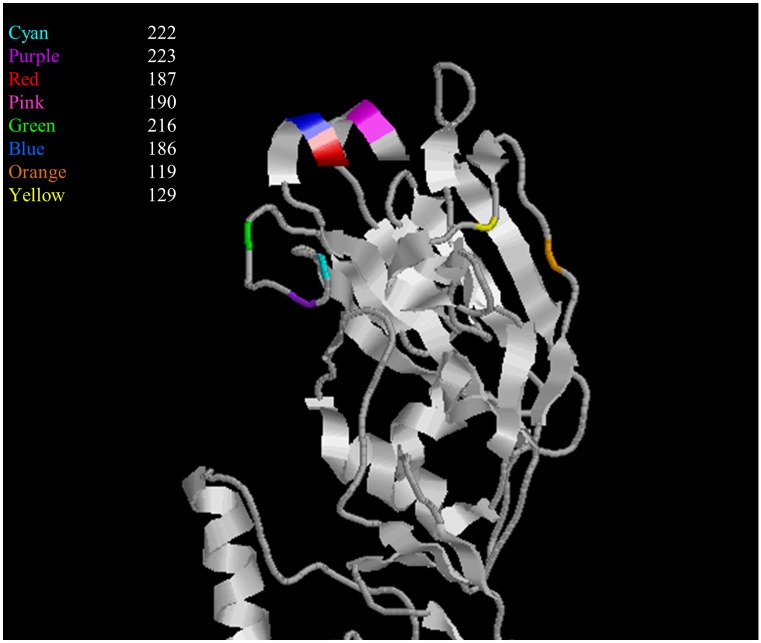
Ribbon trace of globular head region of HA monomer shown as a side view, with the amino acids of interest highlighted. HA residue 222 is shown in Cyan, residue 223 is shown in purple, residue 187 is shown in red, residue 190 is shown in pink, residue 216 is shown in green, residue 186 is shown in blue, residue 119 is shown in orange and residue 129 is shown in yellow.

Previous studies on A(H1N1)pdm09 viruses indicate HA substitutions D222N and Q223R to be egg-adapting changes with the DQ sequence being present in non-egg-adapted viruses. A/Christchurch/16/2010 is antigenically similar to A/California/07/2009, the current WHO recommended virus for vaccine, and the CVV NIB-74xp was prepared from it. For this study, the Chr/16 HA was engineered to contain the non-egg-adapted DQ motif, and substitutions at positions 222, 186, 187, 190 and 216 were introduced into the HA (Table 1). We showed that the non-egg adapted Chr/16 HA was able to stably egg adapt through substitutions D187S, D187V, S190R or I216R (Table 2). The positions of the various residues altered in the study are shown in Fig 3.

A mutation previously shown to be responsible for improved virus yield in an H1N1pdm09 CVV, K119N, could not be rescued with a PR8 backbone without the virus gaining additional mutations in the HA, but could be obtained with a Cal/7 backbone; however, a virus with a K119N/S190R double substitution conferred egg adaptation with either set of internal genes (Table 1). This demonstrates that the combination of backbone and HA sequence may be important for egg adaptation via residue 119 in Chr/16.

Viruses with N129D or A186D required an initial cell passage prior to successful growth in eggs and probably egg adapted via a subsequent Q223R substitution. These findings concur with other studies where 129D and 186D were found in H1N1pdm09 viruses already egg adapted at 222/223 [1,11,12] or 191 [12, 13], and we conclude that (like reside 119) residues 129 and 186 are not independent egg adaptation loci for A/Christchurch/16/2010.

The consistently higher protein and HA yields of all PR8 backbone viruses compared to wt Chr/16 was undoubtedly due to the inclusion of highly egg adapted PR8 internal genes, which is well established as the best way (to date) to improve the yield of wild type viruses for vaccine production in eggs [14,15,16]. There was little significant difference between viruses that included the PR8 internal genes; the dramatic improvement caused by the PR8 internal genes probably masked the more subtle effects on yield of specific amino acid substitutions in the HA gene. In contrast, the effect of the HA amino acid substitutions in viruses bearing A/California/7/2009 internal genes on protein and HA yield was more pronounced.

The data presented here suggest that a D222N substitution in A(H1N1)pdm09 viruses impacts both egg adaptation and yield, since viruses with the same Cal/7 backbone and an alternative egg adaptation substitution generally had poorer yields. However, amongst the 222D/223Q viruses with Cal/07 backbones, the yield was variable and one virus (NIBRG-264; DQ/D187V) had similar yield to NIBRG-260 (NQ). This is possibly due to altered receptor binding involving residue 187; it has been reported that a DQ (non-egg adapted) motif preferentially binds α2,6 linked glycans mediated through residues 187D and 222D [17,18,12,19].

The nature of the 187 residue is also important for yield, since only 187V (NIBRG-264) and not 187S (NIBRG-261) showed improved yield with a Cal/07 backbone. It has been reported that negatively charged residues in A(H1N1)pdm09 HA possibly decrease HA-sialic acid interaction and facilitate release of progeny for multiple cycle replication, which may increase the yield of virus [20]; however, in this study viruses with better yields had either a positively charged (I216R) or a hydrophobic (D187V) substitution. The two poorer yielding viruses had S190R, or an uncharged D187S substitution.

This study has identified various HA residues in addition to 222 and 223 associated with the egg-adaptation of an H1N1pdm09 virus. These alternative residues cluster around the receptor binding site and include residues (and equivalently positioned loci) identified in the egg adaptation of pre-2009 H1N1 viruses. Importantly, these egg-adapting substitutions do not affect the antigenicity of the virus. Egg-adapting substitutions reduce the HA content in viruses with A(H1N1)pdm09 internal genes although in many cases there is an overall increase in yield of total viral and HA protein. In viruses with PR8 internal genes, the effect of egg-adapting substitutions on yield is less pronounced. Nonetheless, data from this study will enhance the assessment of A(H1N1)pdm09 wild-type strains for CVV preparation by widening the choice of isolates that potentially have improved growth and yield in eggs. The virus with one particular substitution, I126R (NIBRG-259), outperformed all other viruses studied, even although the yield enhancement was not statistically significantly different from two other viruses. However, the absolute yield of total viral protein (12.5 mg/100 eggs) would have highlighted this virus as the best CVV of the viruses studied since during CVV development for seasonal vaccine, minimal (i.e. non statistically valid) studies on protein and/or antigen yield are performed due to time constraints. Thus when a future drifted antigenic variant A(H1N1)pdm09 is identified for inclusion in vaccine, egg-adapted viruses with an I126R substitution should be considered as prime candidates for CVV development.
